# Rescue of recombinant peste des petits ruminants virus: creation of a GFP-expressing virus and application in rapid virus neutralization test

**DOI:** 10.1186/1297-9716-43-48

**Published:** 2012-06-02

**Authors:** Qianqian Hu, Weiye Chen, Kehe Huang, Michael D Baron, Zhigao Bu

**Affiliations:** 1 College of Veterinary Medicine, Nanjing Agricultural University, Nanjing, 210095, People’s Republic of China; 2 The Key Laboratory of Zoonoses of Chinese Academy of Agricultural Sciences, Key Laboratory of Veterinary Public Health of Ministry of Agriculture, State Key Laboratory of Veterinary Biotechnology, Harbin Veterinary Research Institute of Chinese Academy of Agricultural Sciences, Harbin, 150001, People’s Republic of China; 3 Institute for Animal Health, Pirbright Laboratory, Pirbright, Surrey, GU24 ONF, United Kingdom

## Abstract

Peste des petits ruminants virus (PPRV) causes high mortality in goats and sheep and the disease has shown a greatly increased geographic distribution over the last 15 years. It is responsible for serious socioeconomic problems in some of the poorest developing countries. The ability to create recombinant PPRV would provide a useful tool for investigating the biology of the virus and the pathology of disease, as well as for developing new vaccines and diagnostic methods. Here we report the first successful rescue of recombinant PPRV from a full-length cDNA clone of the virus genome. Successful recovery of PPRV was achieved by using a RNA polymerase II promoter to drive transcription of the full-length virus antigenome. We have used this technique to construct a virus expressing a tracer protein (green fluorescent protein, GFP). The recombinant virus replicated as well as the parental virus and could stably express GFP during at least 10 passages. The newly established reverse genetics system for PPRV provides a novel method for constructing a vaccine using PPRV as a vector, and will also prove valuable for fundamental research on the biology of the virus. We found that our recombinant virus allowed more rapid and higher throughput assessment of PPRV neutralization antibody titer via the virus neutralization test (VNT) compared with the traditional method.

## Introduction

Peste des petits ruminants (PPR) is a highly contagious disease of domestic and wild small ruminants caused by peste des petits ruminants virus (PPRV); it is responsible for serious socioeconomic problems in some of the poorest developing countries [1-3]. PPR is a notifiable disease listed by the World Organisation for Animal Health (OIE). PPR was first reported in the Ivory Coast in 1942, and later found in the Middle and Near East, southwest and central Asia [4-6], and recently in China [7]. PPRV, which is a member of the genus *Morbillivirus* belonging to the family *Paramyxoviridae*[8], is a linear, non-segmented, single stranded, negative-sense RNA virus with a genome length of 15948 bp. The PPRV genome encodes six structural proteins (nucleocapsid (N), phosphoprotein (P), matrix (M), fusion (F), hemagglutinin (H), and polymerase (L)), and two nonstructural proteins (C and V), which are in the order of 3′-N-P/C/V-M-F-H-L-5′ on the genome [9-12]. PPRV vaccine strain (Nigeria 75/1, PPRV/N75/1) has been widely used as a safe and efficacious live vaccine to control PPR infections [13].

Several studies have indicated that recombinant paramyxoviruses are effective and genetically stable vectors with many advantages [14-16] due to their relatively simple reverse genetic systems. However, a reverse genetic system for PPRV has so far not been possible, despite effort in several laboratories, although a rescue system for rinderpest virus (RPV), which is evolutionary closest to PPRV, has been known since 1997 [17], and a PPRV mini-genome rescue system was described in 2007 [18].

Green fluorescent protein (GFP) is a useful tracer protein to observe and optimize virus rescue efficiency, and to study the characteristics of rescued viruses. Recombinant viruses expressing GFP could also be utilized to genetically mark vaccines to allow serological differentiation between animals that have been vaccinated against PPR and those recovering from natural infection [16,19] or to improve the virus neutralization test (VNT) [20-23]. In this study, we developed a system for recovering recombinant PPRV and introduced the GFP open reading frame into a recombinant form of PPRV/N75/1 to create a marked recombinant PPRV which we have used initially to improve PPRV VNT assays for use in studies of immune responses to different vaccines.

## Materials and methods

### Cells and viruses

PPRV vaccine strain (Nigeria 75/1, PPRV/N75/1) was obtained from the China Institute of Veterinary Drug Control, Beijing, China. Vero cells (The American Type Culture Collection, ATCC: CCL-81) were cultured in Dulbecco’s modified Eagle’s medium (DMEM) (Gibco, Carlsbad, CA, USA) containing 10% fetal bovine serum (FBS) (Gibco). Vero cells expressing the canine form of the general morbillivirus receptor, signaling lymphocyte activation molecule (SLAM), (VDS cells) were the kind gift of Dr P Duprex, Queen’s University Belfast, UK and were maintained in DMEM/10% FBS/0.1 mg/mL Zeocin. PPRV/N75/1 or rescued recombinant PPRV were propagated and titrated in Vero cells cultured in DMEM containing 2% FBS.

### Serum samples

Ten goats (nos. 1–10) and 10 sheep (nos. 11–20) were vaccinated twice with 2 × 10^5^ 50% tissue culture infective dose (TCID_50_) of recombinant capripoxvirus (rCPV) expressing PPRV glycoprotein H (rCPV-PPRVH) [24] with a three-weeks interval. Serum samples were collected two weeks following the second vaccination. Eleven additional goats (nos. 21–31) were vaccinated with 10^7^ TCID_50_ PPRV/N75/1, and serum samples were collected four weeks post-vaccination. Serum samples were collected from each test animal before vaccination to act as negative controls.

### Plasmid construction

**Figure 1:**
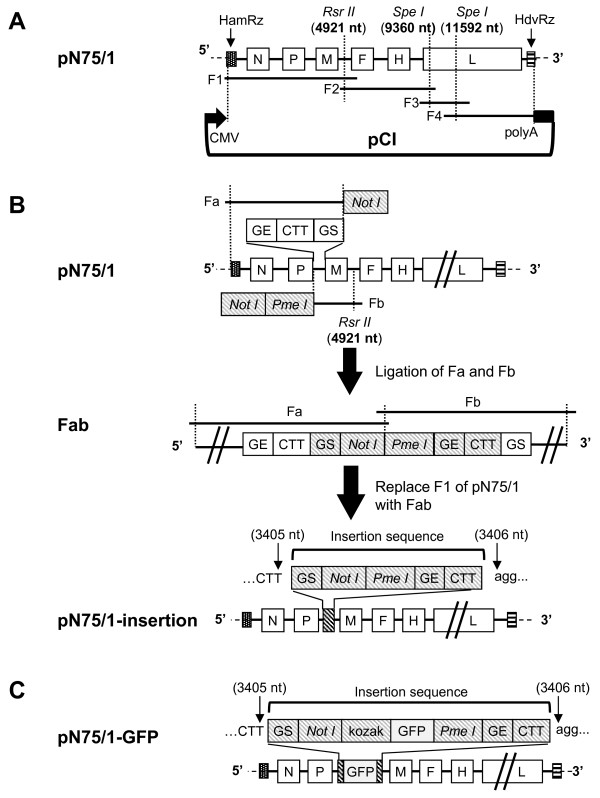
**Construction of plasmids for PPRV rescue.** (**A**) The cDNA fragments F1, F2, F3 and F4 were reverse transcribed and amplified from PPRV/N75/1 genomic RNA. The hammerhead ribozyme sequence (HamRz) and the hepatitis delta virus ribozyme sequence (HdvRz) were introduced to the 5′ end of F1 and the 3′ end of F4, respectively. All fragments were then subcloned stepwise into the pCI vector to produce plasmid pN75/1. (**B**) DNA fragments Fa (from the HamRz to the GS sequence of M with a *Not I* site introduced at 3′ end) and Fb (from GE of P gene to the end of F1 with *Not I* and *Pme* I sites introduced at the 5′ end) were PCR-amplified from pN75/1 and ligated together to get fragment Fab, then section F1 of pN75/1 was replaced with Fab to get plasmid pN75/1-insertion. The net result was equal to insertion of a morbillivirus gene start (GS) sequence, *Not* I and *Pme* I sites, gene end (GE) sequence and CTT intergenic trinucleotide into pN75/1 between nt 3405 and 3406 of the PPRV/N75/1 genome cDNA sequence. (**C**) The GFP ORF with a Kozak sequence at the 5′ end of the ORF was inserted into plasmid pN75/1-insertion to produce plasmid pN75/1-GFP.

PPRV/N75/1 was propagated in Vero cells, and RNA from infected cells isolated. The entire viral genome was amplified by RT-PCR using high-fidelity *Pfx* DNA polymerase (Invitrogen, Carlsbad, CA, USA) in four overlapping sections (F1 to F4), which were assembled into a full-length cDNA clone (Figure 1a). The complete cDNA of the genome of the virus stock used was fully sequenced and confirmed [GenBank: HQ197753]. A number of minor differences between the sequence determined for this stock of the PPRV/N75/1 vaccine and that previously published for PPRV/N75/1 [GenBank: X74443] were noted, but it is not possible to tell if these are due to mistakes in the earlier sequence or to changes to the vaccine seed stock over time. The hammerhead ribozyme sequence (HamRz) and hepatitis delta virus ribozyme sequence (HdvRz) were introduced at the 5′ and 3′ ends of the antigenomic sequence, respectively, as previously described by Inoue et al. [25,26]. The assembled HamRz-(full-length genomic cDNA)-HdvRz was then cloned into the pCI vector (Promega, Madison, WI, USA) under the control of the CMV promoter. The resulting plasmid was named pN75/1 (Figure 1a).

New gene fragments were then inserted into pN75/1 between nt 3405 and 3406 of the PPRV/N75/1 sequence (i.e. at the start of the M gene) as illustrated in Figure 1b: a 32 nt gene start (GS) sequence (5′-aggagcaagggcaactgagcttcacagacaag-3′), a *Not* I restriction site, a *Pme* I restriction site, a 66 nt gene end (GE) sequence (5′-cacatcctataatcaacatctcatactcggttgaaaacatcctctcaatcaggctattacaaaaaa-3′) and a CTT intergenic trinucleotide. In brief, the genome construction was carried out as follows: DNA fragment Fa (ending at the GS of the M gene with a *Not I* site introduced at 3′ end) and Fb (starting from the GE of the P gene with *Not I* and *Pme* I sites introduced at the 5′ end) were PCR-amplified from pN75/1 and ligated together to get DNA Fab, then DNA F1 used in the original construction of pN75/1 was replaced with DNA Fab to give plasmid pN75/1-insertion (Figure 1b). The net result was equal to insertion of the five genetic elements above into pN75/1 between nt 3405 and 3406 of the PPRV/N75/1 genome cDNA sequence.

Finally, we inserted the open reading frame (ORF) for GFP into pN75/1-insertion. The GFP ORF was PCR-amplified from pIRES2-EGFP (Clontech, Mountain View, CA, USA) using PrimeSTAR HS DNA polymerase (Takara, Shiga, Japan) with primers 5′-tctgcggccgc**gccgccacc**atggtgagcaagggcgag-3′ (the *Not I* site is underlined and the Kozak sequence is in bold) and 5′-ctcgtttaaacttacttgtacagctcgtc-3′ (the *Pme I* site is underlined). The amplified product was ligated into *Eco*R *V*-cut pBluescript II KS(+), removed from that plasmid by digestion with *Not I* and *Pme I* and cloned into pN75/1-insertion, cut with the same enzymes, to produce plasmid pN75/1-GFP (Figure 1c).

In addition, the ORFs of the N, P and L genes were amplified from pN75/1 and inserted into pCAGGS to construct helper plasmids. The resultant plasmids were named pCA-N, pCA-P and pCA-L, respectively. All primer sequences used in this study are available from the corresponding author upon request.

### Transfection of Vero cells and rescue of recombinant viruses from cloned cDNA

To rescue the recombinant PPRV or PPRV/GFP, 90% confluent Vero or VDS cells in one well of a 6-well plate were transfected with the plasmids pCA-N (2 μg), pCA-P (1 μg) and pCA-L (1 μg) together with 4 μg of pN75/1-GFP. Lipofectamine 2000 (Invitrogen) or TransIt-LT1 (Mirus Biologicals) were used for transfections following the manufacturers’ instructions. After 7–9 days of incubation at 37°C, the cells and supernatants were collected and freeze-thawed twice and then passaged in fresh cells to propagate the rescued virus. Supernatants from cytopathic effect-positive wells were used to propagate viral stocks in Vero or VDS cells. The complete genomic sequences of the rescued viruses were confirmed by sequencing. The rescued viruses were named rPPRV/N75/1 and rPPRV/GFP.

### Growth curves

Vero cells were grown to 70% confluence in 6-well plates and infected with 0.1 multiplicity of infection (MOI) of virus for 1 h. The inoculum was removed and the cells were washed twice with medium, then 2 mL of medium were added to each well. Cells with medium were stored at −70°C each day from 3 to 8 days post-infection, and were freeze-thawed twice before titration. The TCID_50_ of released virus was quantitated by established methods [27].

### Immunofluorescence assays

Vero-SLAM cells grown in 12-well plates on glass coverslips were infected with PPRV/N75/1 or rPPRV/GFP at a MOI of 0.05 and incubated for 2 d. Cells were fixed with 3% paraformaldehyde in PBS and stained with monoclonal antibody recognizing PPRV H followed by AlexaFluor 568-labelled anti-mouse IgG (Invitrogen). Cells were stained with DAPI for 5 min before mounting to stain the nuclei of all cells. Images were taken by sequential scanning at each wavelength on a Leica confocal microscope. Mock-infected cells were used as controls. For live cell imaging, Vero cells were infected at a MOI of 0.1 and imaged at the indicated time post infection using an inverted fluorescence microscope (Zeiss, Oberkochen, Germany).

### Western blotting

Vero cells were infected with PPRV/N75/1 or rPPRV/GFP at a MOI of 0.1 and incubated until the cytopathic effect (CPE) involved 60–80% of cells. The cell extracts were then analyzed by SDS-PAGE and blotted on a nitrocellulose membrane. The membrane was incubated with mouse anti-GFP monoclonal antibody (Sigma) as a primary antibody, and with peroxidase-conjugated goat anti-mouse IgG (Sigma) as a secondary antibody. Immunostained proteins were visualized using 3,3′-diaminobenzidine reagent. Mock-infected Vero cells were used as controls.

### Analysis of GFP fluorescence

Vero cells grown in 6-well plates were infected with rPPRV/GFP at a MOI of 0.1. The cells together with their medium were freeze-thawed twice when the CPE reached 100%, and 200 μL of this broken cell preparation was added to 100 μL of cell-lysis buffer (0.15 M Tris-Cl, pH 8.0, 1.5% Triton X-100). After incubating for 15 min, 100 μL aliquots of cell lysate were transferred to wells of a 96-well white plate (Corning, Lowell, MA, USA). Mock-infected cells were used as controls. The GFP fluorescence of each well was read on a Microplate Fluorescence Reader (Bio-Tek, Winooski, VT, USA). The excitation peak was set at 485 nm, the emission peak at 528 nm, and the sensitivity at 50. The relative fluorescence units (RFU) were calculated as: [(fluorescence of the test well) - (fluorescence of the control well)].

### Virus neutralization tests

Titrating of PPRV-neutralizing antibody (VNA) in serum samples were performed in quadruplicate in 96-well plates as previously described [24] following OIE recommendations [28]. All serum samples were inactivated by heating at 56°C for 30 min before testing. The inactivated sera were diluted five-fold in triplicate, and then serially diluted two-fold for VNA titration. PPRV/N75/1 or rPPRV/GFP (100 TCID_50_ in 100 μL cell culture medium) was mixed with 100 μL of diluted serum in a 96-well plate and incubated at 37°C for 1 h. Vero cells (50 μL) were added to each well and the plates were incubated at 37°C. The CPE was recorded at day 14 for PPRV/N75/1 as described previously [28]. A titer ≥ 10 was considered positive.

### Statistical analysis

The statistical analyses of the comparison between the results of assays using PPRV/75/1 and rPPRV/GFP were carried out using a paired *t* test as calculated using the GraphPad Prism program. A P value < 0.05 was considered significant.

## Results

### Rescue of rPPRV and GFP expression of rPPRV/GFP in vitro

Previous attempts to rescue PPRV from full-length copies of the genome had used T7 RNA polymerase-driven transcription of the virus antigenome, since this method has been successful with all other morbilliviruses, indeed most viruses of the order *Mononegavirales*, rescued to date. We hypothesised that the PPRV rescue may not have worked because of some sequence element in the PPRV genome (e.g. cryptic transcription termination signals) that were preventing full genome synthesis. We therefore attempted the recovery of recombinant PPRV using an RNA pol II promoter to drive transcription, and ribozymes at both ends of the PPRV sequence to ensure that the final transcript had exact viral termini, as has been shown to be effective for rabies virus rescue [25]. We therefore constructed the PPRV genome plasmid as described in Methods (Figure 1) as well as appropriate helper plasmids expressing the N, P and L proteins of PPRV, and rPPRV/N75/1 and rPPRV/GFP were rescued successfully on Vero cells. Because rPPRV/GFP is much easier than rPPRV/N75/1 for evaluating or optimizing the reverse genetic system, rPPRV/GFP was used in all following experiments. Optimum ratios of plasmids were determined based on the frequency of GFP-positive cells after transfection, and we were able to recover recombinant PPRV/GFP in 50%–80% of transfected wells using the final method as described.

**Figure 2:**
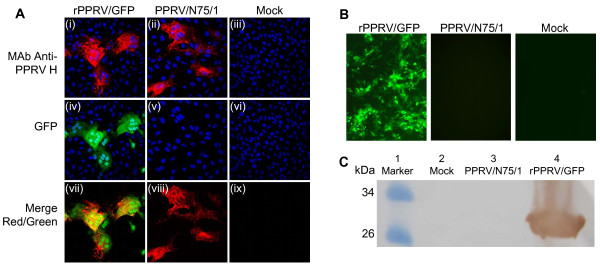
**Replication of and GFP expression by rPPRV/GFP in Vero cells.** (**A**) Cells infected with either rPPRV/GFP (i, iv, vii) or PPRV/N75/1 (ii, v, viii), or mock infected (iii, vi, ix), were fixed and labeled for the presence of PPRV glycoprotein H (red); GFP was detected by its natural fluorescence. Cells were counterstained with DAPI to show the nuclei. (**B**) GFP expression in live, PPRV/N75/1- and rPPRV/GFP-infected Vero cells was observed by direct observation of unfixed cells 2 days post infection. (**C**) Lysates of Vero cells that had been infected with PPRV/N75/1 or rPPRV/GFP were analyzed by Western blotting using mouse anti-GFP monoclonal antibody. Mock-infected Vero cells were used as a controls.

To confirm rPPRV/GFP replication and normal viral protein expression as well as GFP expression, infected VDS cells were immunostained with anti-PPRV H MAb as described in Methods. Immunofluorescence microscopy showed clear labeling of cells infected with either PPRV/N75/1 or rPPRV/GFP (Figure 2a), while only cells infected with rPPRV/GFP showed the green fluorescence expected of GFP expression (Figure 2a). The GFP expression was strong enough to be seen even in early stages of infection, as shown by the appearance of cells at the borders of infection foci which were clearly green but had not yet expressed detectable amounts of the viral H protein. Infected cells could easily be seen by live cell imaging (Figure 2b). The expression of GFP was further confirmed by Western blot analysis, which showed that a protein of the appropriate size was detected with anti-GFP antibody in lysates of rPPRV/GFP-infected cells (Figure 2c, lane 4), while no band was detected with mock- or PPRV/N75/1-infected cells (Figure 2c, lanes 2 and 3).

### Virus growth and stability in vitro

**Figure 3:**
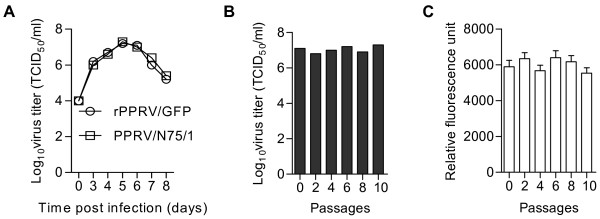
**Growth and stability of rPPRV/GFP in vitro.** (**A**) Vero cells were infected with PPRV/N75/1 or rPPRV/GFP, and the amount of virus in the cultures was measured at various times from 0 to 8 d post-infection. (**B**) rPPRV/GFP was passaged in Vero cells every 6–7 d, and culture medium and cells of each passage was freeze-thawed twice and used for virus titration (**B**) and to analyze GFP expression by quantitating relative fluorescence (**C**). Data are expressed as the mean ± standard deviation (SD).

To determine whether the rescue procedure or exogenous gene insertion affected the replicative ability of our recombinant virus, growth curves of rPPRV/GFP and PPRV/N75/1 in infected Vero cells were determined. The results (Figure 3a) show that there was no discernible difference in growth rate or maximum titre between the two viruses. To determine whether repeated passage of rPPRV/GFP in Vero cells affected replication and expression, rPPRV/GFP was propagated in Vero cells for 10 passages, and samples of culture medium plus cells collected every second passage; these samples were assayed for total (cell associated and medium) virus titre and for GFP expression. The results show that the virus titer (Figure 3b) and GFP expression (Figure 3c) from different passages changed only slightly.

### PPR virus neutralization test using rPPRV/GFP

**Figure 4:**
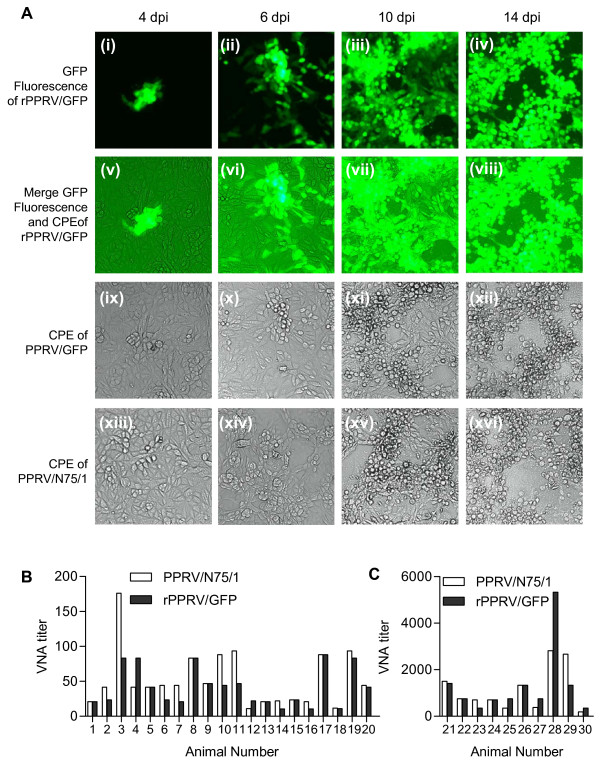
**Comparison of PPR virus neutralization test using rPPRV/GFP and PPRV/N75/1.** (**A**) Detection of virus replication during VNT assay. Serum from a goat (no. 31) previously vaccinated with 10^7^ TCID_50_ PPRV/N75/1 was tested using either rPPRV/GFP or PPRV/N75/1. The GFP fluorescence ((i) to (iv)) and CPE ((ix) to (xii)) of rPPRV/GFP or the CPE of PPRV/N75/1 ((xiii) to (xvi)) after treatment with diluted serum (320-fold) were observed at 4, 6, 10 and 14 d post-infection. (**B**) Twenty PPRV-positive sera from rCPV-PPRVH vaccinated goats (nos. 1–10) or sheep (nos. 11–20) were assayed for PPR VNA titer using PPRV/N75/1 and rPPRV/GFP. (**C**) Ten positive sera from PPRV/N75/1 vaccinated goats (nos. 21–30) were assayed in the same manner as above. The results from assays with the two viruses were compared using a paired *t* test, and no significant difference seen

GFP expression from our rPPRV/GFP is easily observed in live cells by fluorescence microscopy. We took advantage of this to help us observe virus growth during the carrying out of VNTs, growth that is normally detected by the observation of cytopathic effect (CPE). Serum from a vaccinated sheep (no. 31) was tested in a VNT assay using rPPRV/GFP or PPRV/N75/1. GFP fluorescence (Figure 4a (i-iv)) and CPE (Figure 4a (ix-xii)) of rPPRV/GFP or CPE of PPRV/N75/1 (Figure 4a (xiii-xvi)) was observed at 4, 6, 10 and 14 d post-infection in wells where virus was incubated with 320-fold diluted serum. The GFP fluorescence could be observed as early as day 4 (Figure 4a (i)), and was very clear after day 6 (Figure 4a (ii-iv)), whereas the PPRV/GFP (Figure 4a (xi-xii)) and PPRV/N75/1 CPE (Figure 4a (xv-xvi)) were only clearly observed from day 10. Therefore, the rPPRV/GFP VNT results were available at least four days earlier than if using PPRV/N75/1.

In order to verify that the two methods gave the same titre, 20 PPRV-positive sera from rCPV-PPRVH vaccinated goats (nos. 1–10) or sheep (nos. 11–20) were assayed for PPR VNA titer using either PPRV/N75/1 or rPPRV/GFP. As shown in Figure 4b, there was no statistically significant difference (*P* > 0.05, paired *t*-test) between the VNA titer results using the two viral strains. Ten additional positive serum samples from PPRV/N75/1-vaccinated goats (nos. 21–30), which have much higher VNA titres, were assayed in the same manner, and with same results (*P* > 0.05, Figure 4c). The VNA titer of 30 (negative) serum samples collected from 30 test animals (nos. 1–30) before vaccination were all lower than 5 (data not shown) using either rPPRV/GFP or PPRV/N75/1.

## Discussion

Here, a full PPRV reverse genetics system has been established for the first time. Since the establishment of a RPV reverse genetics system more than a decade ago [17], several groups have attempted to establish a PPRV reverse genetics system [18,29]. However, the attempts have been unsuccessful to date (personal communication). We have now successfully rescued a recombinant PPRV expressing GFP. Rescue efficiencies were acceptable, but not high, and rescue efficiency may have to be improved to enable recovery of PPRV with more extensive or deleterious mutations, either to use PPRV as a novel vaccine vector or for fundamental research.

Our results showed that neither rescue conditions nor insertion of an additional gene affected recombinant virus replication and passage stability in Vero cells. This is in contrast to the findings with similar recombinant viruses made using the vaccine strain of RPV [19,30], where the insertion of an extra gene between the virus P and M genes led to a reduction in growth rate. GFP expression was high, suggesting potential use of a PPRV reverse genetics system to construct recombinant multivalent vaccines by replacing the GFP ORF with the coding sequence for an immunogenic antigen from another virus, or the creation of tagged viruses for use in fundamental research on the growth and spread of PPRV in its hosts, as has been recently carried out with measles virus [31,32]. A version of rPPRV/GFP could be used as a marker vaccine, especially if GFP is expressed as a membrane-anchored protein, which was necessary with RPV-based constructs to elicit a serum antibody response to the GFP [16,19].

PPRV/N75/1 replicates relatively slowly in Vero cells, with 14 days typically required for observation of CPE in VNT assays where low initial MOIs are used. CPE during early infection (1–6 d post-infection) can be very difficult to observe. In addition, complex serum components may cause CPE-like cell death at dilutions below 20-fold even when the sera are heat-inactivated at 56°C. When the VNA titer is between 5 and 20, it is difficult to judge whether cell death has been caused by virus or by the serum itself, which may lead to false-negative results and underestimation of VNA titre. rPPRV/GFP could easily solve the two above-mentioned problems. First, virus growth can be observed with the help of GFP fluorescence as early as four days post infection in these assays, and VNT results could be determined with confidence as early as six days post infection. Thus, eight days could be saved compared with traditional methods using PPRV/N75/1. Secondly, viral CPE could be distinguished easily from CPE-like cell death caused by serum with the help of GFP fluorescence, even though only a limited number of cells are infected by rPPRV/GFP. There is also the potential to use machine scanning of wells of 96-weel plates in a fluorescence plate reader to automate scoring of VNT assays, allowing higher throughput of samples, an important consideration where large scale trials of vaccines or tests for vaccine effectiveness are taking place.

## Competing interests

ZB and WC are inventors on a pending patent application for the reverse genetics system of PPRV and rPPRV/GFP. Other authors declare no financial or non-financial competing interests in the publication of this work.

## Authors’ contributions

QH and WC carried out all the experiments except confocal microscopy and contributed to the drafting of the manuscript; KH contributed to the cloning and sequencing of PPRV genome RNA; MDB contributed to the construction of the genome plasmid, carried out the confocal microscopy and contributed to the drafting of the manuscript; ZB designed the whole study, provided general supervision and prepared the manuscript. All authors have read and approved the submitted manuscript.
